# Assessing the Utility of Multimodal Large Language Models (GPT-4 Vision and Large Language and Vision Assistant) in Identifying Melanoma Across Different Skin Tones

**DOI:** 10.2196/55508

**Published:** 2024-03-13

**Authors:** Katrina Cirone, Mohamed Akrout, Latif Abid, Amanda Oakley

**Affiliations:** 1 Schulich School of Medicine and Dentistry Western University London, ON Canada; 2 AIPLabs Budapest Hungary; 3 Department of Computer Science University of Toronto Toronto, ON Canada; 4 Department of Dermatology Health New Zealand Te Whatu Ora Waikato Hamilton New Zealand; 5 Department of Medicine Faculty of Medical and Health Sciences The University of Auckland Auckland New Zealand

**Keywords:** melanoma, nevus, skin pigmentation, artificial intelligence, AI, multimodal large language models, large language model, large language models, LLM, LLMs, machine learning, expert systems, natural language processing, NLP, GPT, GPT-4V, dermatology, skin, lesion, lesions, cancer, oncology, visual

## Abstract

The large language models GPT-4 Vision and Large Language and Vision Assistant are capable of understanding and accurately differentiating between benign lesions and melanoma, indicating potential incorporation into dermatologic care, medical research, and education.

## Introduction

Large language models (LLMs), artificial intelligence (AI) tools trained on large quantities of human-generated text, are adept at processing and synthesizing text and mimicking human capabilities, making the distinction between them nearly imperceptible [1]. The versatility of LLMs in addressing various requests, coupled with their capabilities in handling complex concepts and engaging in real-time user interactions, indicates their potential integration into health care and dermatology [1,2]. Within dermatology, studies have found LLMs can retrieve, analyze, and summarize information to facilitate decision-making [3].

Multimodal LLMs with visual understanding, such as GPT-4 Vision (GPT-4V) [4] and Large Language and Vision Assistant (LLaVA) [5], can also analyze images, videos, and speech, a significant evolution. They can solve novel, intricate tasks that language-only systems cannot, due to their unique capabilities combining language and vision with inherent intelligence and reasoning [4,5]. This study assesses the ability of publicly available multimodal LLMs to accurately recognize and differentiate between melanoma and benign melanocytic nevi across all skin tones.

## Methods

Our data set comprised macroscopic images (900 × 1100 pixels; 96-dpi resolution) of melanomas (malignant) and melanocytic nevi (benign) obtained from the publicly available and validated MClass-D data set [6], Dermnet NZ [7], and dermatology textbooks [8]. Each LLM was provided with 20 unique text-based prompts that were each tested on 3 images (n=60 unique image-prompt combinations) consisting of questions about “moles” (the term used for benign and malignant lesions), instructions, and image-based prompts where the image was annotated to alter the focus. Our prompts represented potential users, such as general physicians, providers in remote areas, or educational users and residents. The chat content was deleted before each submitted prompt to prevent repeat images influencing responses, and testing was performed over a 1-hour timespan, which is insufficient for learning to take place. Prompts were designed to either involve conditioning of ABCDE (asymmetry, border irregularity, color variation, diameter >6 mm, evolution) melanoma features or to assess effects of background skin color on predictions. Conditioning involved asking the LLM to differentiate between benign and malignant lesions where one feature (eg, symmetry, border irregularity, color, diameter) remained constant in both images to determine whether the fixed element was involved in overall reasoning. To assess the impact of color on melanoma recognition, color distributions of nevi and melanoma were manipulated by decolorizing images or altering their colors.

## Results

Analysis revealed GPT-4V outperformed LLaVA in all examined areas, with overall accuracy of 85% compared to 45% for LLaVA, and consistently provided thorough descriptions of relevant ABCDE features of melanoma (Table 1 and Multimedia Appendix 1). While both LLMs were able to identify melanoma in lighter skin tones and recognize that dermatologists should be consulted for diagnostic confirmation, LLaVA was unable to confidently recognize melanoma in skin of color nor comment on suspicious features, such as ulceration and bleeding.

**Table 1:** Performance of Large Language and Vision Assistant (LLaVA) and GPT-4 Vision (GPT-4V) for melanoma recognition.

Feature		LLaVA	GPT-4V
Melanoma detection		Melanoma identified—referenced shape and color	Melanoma identified—referenced the other ABCDEs^a^ of melanoma
**Feature conditioning**			
	Asymmetry	Melanoma identified—referenced size and color	Melanoma identified—referenced the other ABCDEs of melanoma
	Border irregularity	Melanoma identified—referenced size and color	Melanoma identified—referenced the other ABCDEs of melanoma
	Color	Melanoma identified—incorrectly commented on color distribution	Melanoma identified—referenced the other ABCDEs of melanoma
	Diameter	Melanoma missed—confused by the darker color	Melanoma identified—referenced the other ABCDEs of melanoma
	Color + diameter	Melanoma missed—confused by the darker color and morphology	Melanoma identified—referenced morphology, complexity, color, and border
	Evolution	Melanoma identified—referenced size and color	Melanoma identified—referenced the other ABCDEs of melanoma
**Color bias**			
	Benign—darkened pigment	Darkened lesion classified as melanoma, became confused about other melanoma features	Darkened lesion classified as melanoma, became confused about other melanoma features
	Melanoma—darkened pigment	Darkened lesion classified as melanoma, became confused about the other ABCDEs of melanoma	Darkened lesion classified as melanoma, became confused about the other ABCDEs of melanoma
	Melanoma—lightened pigment	Unable to recognize malignancy and to identify that the image had been altered	Melanoma identified—referenced the other ABCDEs of melanoma and recognized that the altered image had been lightened
**Skin of color**			
	Melanoma detection	Diagnostic uncertainty—unsure of lesion severity and diagnosis	Melanoma identified—referenced the other ABCDEs of melanoma
	Suspicious features	Did not identify suspicious features	Identified suspicious features and recommended medical evaluation—ulceration, bleeding, and skin distortion
**Image manipulation**			
	Visual referring	Tricked into thinking the annotations indicated sunburned skin	Correctly identified that the annotations were artificially added and could be used to monitor skin lesion evolution or to communicate concerns between providers
	Rotation	Tricked into thinking an altered image orientation constituted a novel image	Correctly indicated it could not differentiate between the 2 images and accurately referenced the ABCDEs of melanoma

^a^ABCDE: asymmetry, border irregularity, color variation, diameter >6 mm, evolution.

## Discussion

**Figure 1:**
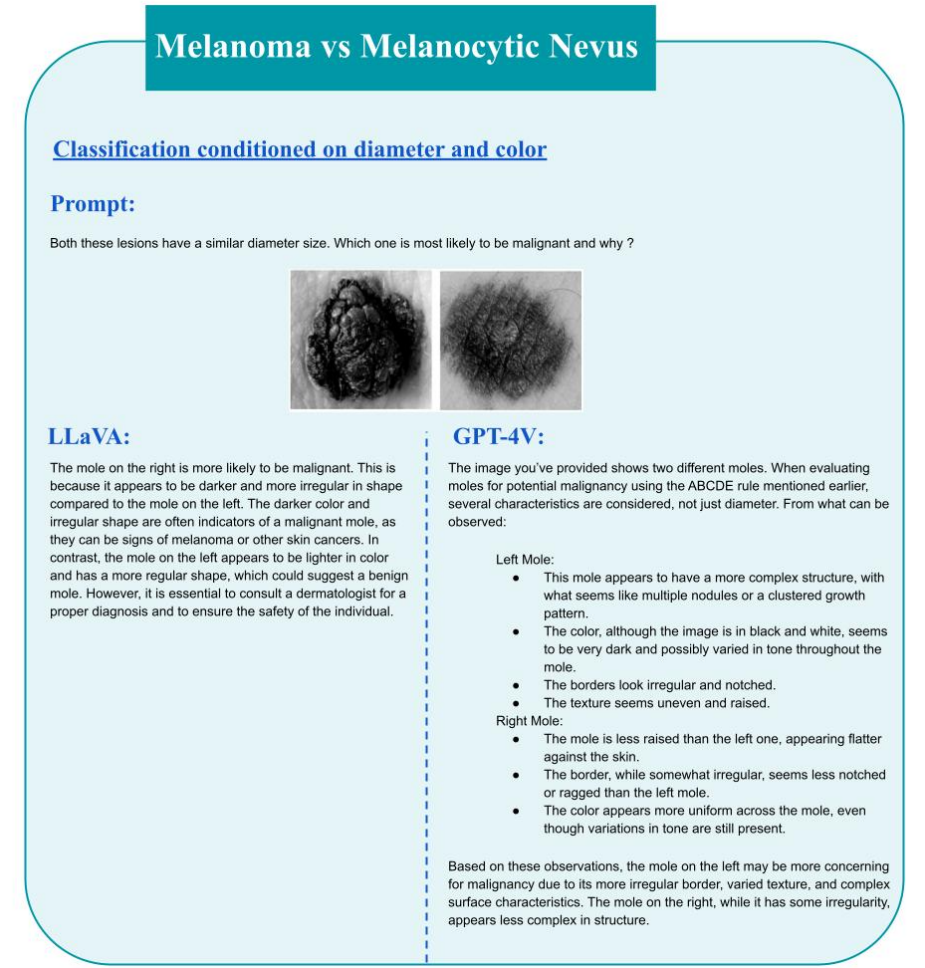
Melanoma detection when conditioned on color and diameter. GPT-4V: GPT-4 Vision; LLaVA: Large Language and Vision Assistant.

Across all prompts analyzing feature conditioning, GPT-4V correctly identified the melanoma, while LLaVA did not, when color, diameter, or both were held constant (Figure 1). This suggests these features influence melanoma detection in LLaVA, with less importance placed on symmetry and border. Both LLMs were susceptible to color bias, as when a pigment was darkened with all other features held constant, the lesion was believed to be malignant. Alternatively, when pigments were lightened, GPT-4V appropriately recognized this alteration, while LLaVA did not. Finally, image manipulation did not impact GPT-4V’s diagnostic abilities; however, LLaVA was unable to detect these manipulations and was vulnerable to visual referring associated with melanoma manifestations. The red lines added around the nevus’s edges were identified as sunburned skin when presented to LLaVA, while GPT-4V correctly recognized these annotations as useful for monitoring lesion evolution or communicating specific concerns between health care providers.

Although limitations are present, GPT-4V can accurately differentiate between benign and melanoma lesions. Performing additional training of these LLMs on specific conditions can improve their overall performance. Despite our findings, it is critical to account for and address limitations such as reproduction of existing biases, hallucinations, and visual prompt injection vulnerabilities and incorporate validation checks before clinical uptake [9]. Recently, the integration of technology within medicine has accelerated, and AI has been used in dermatology to augment the diagnostic process and improve clinical decision-making [10]. There is an urgent global need to address high volumes of skin conditions posing health concerns, and the integration of multimodal LLMs, such as GPT-4V, into health care has the potential to deliver material increases in efficiency and improve education and patient care.

## Appendix

Multimedia Appendix 1 The 20 unique text-based prompts provided to GPT-4 Vision and Large Language and Vision Assistant and the responses of both large language models depicted side by side.

## Abbreviations

ABCDE: asymmetry, border irregularity, color variation, diameter >6 mm, evolution
AI: artificial intelligence
GPT-4V: GPT-4 Vision
LLaVA: Large Language and Vision Assistant
LLM: large language model
