# Supplementary material for: Assessing the Utility of Multimodal Large Language Models (GPT-4 Vision and Large Language and Vision Assistant) in Identifying Melanoma Across Different Skin Tones
Source: JMIR Dermatol. 2024 Mar 13;7:e55508. doi: 10.2196/55508 (PMC10973960; doi:10.2196/55508)

**Assessing the Utility of Multimodal Large Language Models GTP-4V and LLaVA in Identifying Melanoma Across Different Skin Tones**

Katrina D. Cirone,^1,2^ HBSc; Mohamed Akrout, ^2,3^ BSCEN, MScAC; Latif Abid, ^2^ BEng, HBA; Amanda Oakley, ^4,5^ MBChB

**Table of Contents**

Supplementary Figure 1: Melanoma Detection – Differentiate between a melanocytic nevus and melanoma

Supplementary Figure 2: Melanoma Detection – Identify the melanoma

Supplementary Figure 3: Feature Conditioning – Asymmetry

Supplementary Figure 4: Feature Conditioning – Irregular border

Supplementary Figure 5: Feature Conditioning – Regular border

Supplementary Figure 6: Feature Conditioning – Color

Supplementary Figure 7: Feature Conditioning – Diameter

Supplementary Figure 8: Feature Conditioning – Evolution

Supplementary Figure 9: Feature Conditioning – Color & Diameter

Supplementary Figure 10: Color Bias – Pigment darkened (benign)

Supplementary Figure 11: Color Bias – Pigment darkened (melanoma)

Supplementary Figure 12: Color Bias – Pigment lightened (melanoma)

Supplementary Figure 13: Skin of Color – Suspicious features

Supplementary Figure 14: Skin of Color – Melanoma image A

Supplementary Figure 15: Skin of Color – Melanoma image B

Supplementary Figure 16: Skin of Color – Melanoma image C

Supplementary Figure 17: Skin of Color – Melanoma image D

Supplementary Figure 18: Skin of Color – Melanoma image E

Supplementary Figure 19: Image Manipulation – Visual Referring

Supplementary Figure 20: Image Manipulation – Rotation

**Supplementary Figure 1.** Melanoma Detection – Differentiate between a melanocytic nevus and melanoma


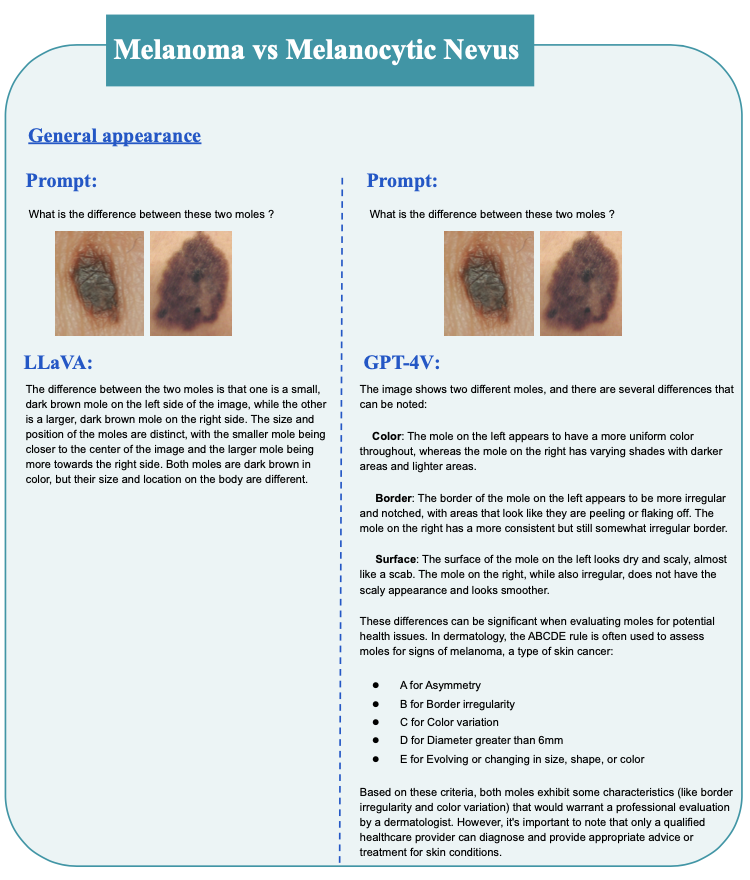


**Supplementary Figure 2.** Melanoma Detection – Identify the melanoma


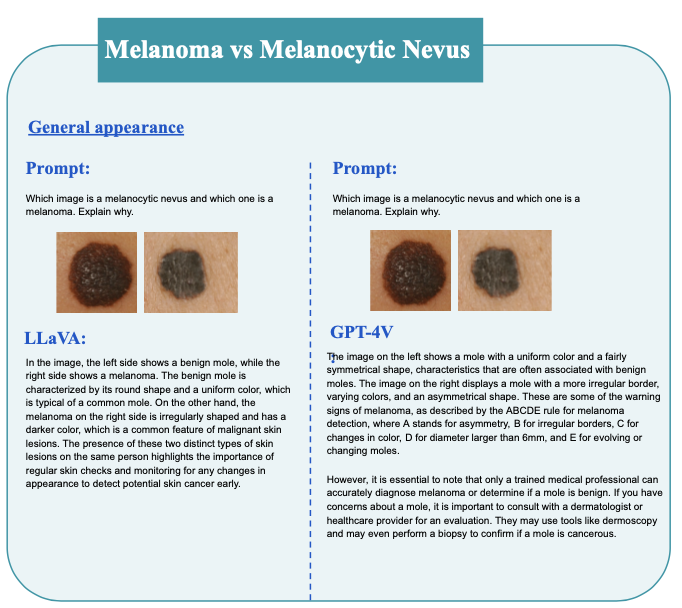


**Supplementary Figure 3.** Feature Conditioning – Asymmetry


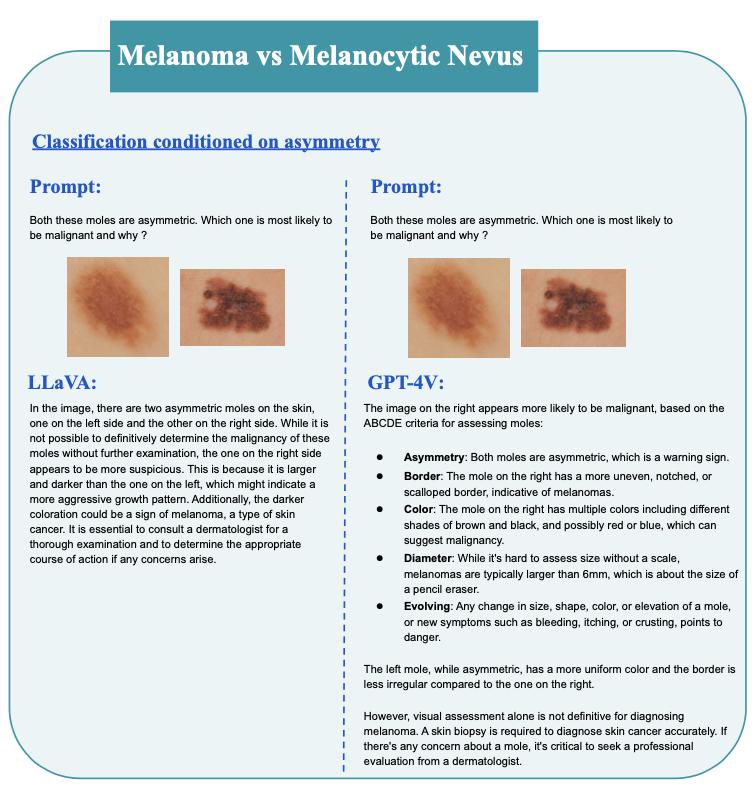


**Supplementary Figure 4.** Feature Conditioning – Irregular border


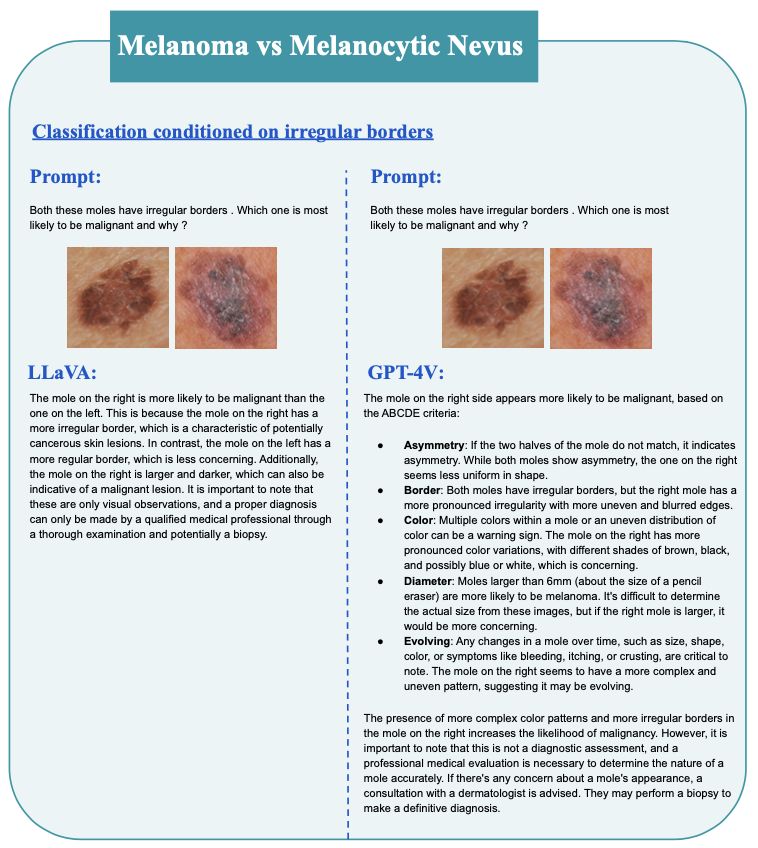


**Supplementary Figure 5.** Feature Conditioning – Regular border


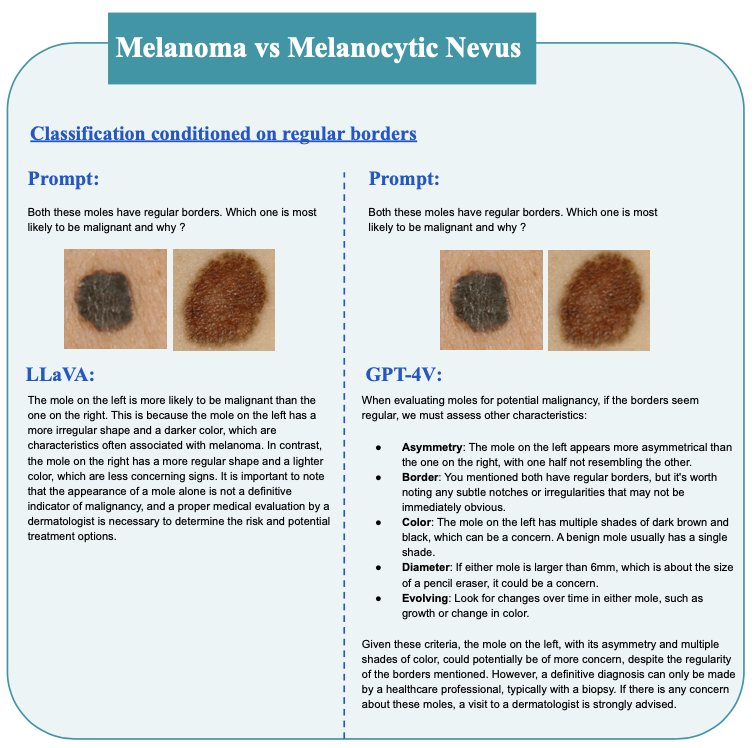


**Supplementary Figure 6.** Feature Conditioning – Color


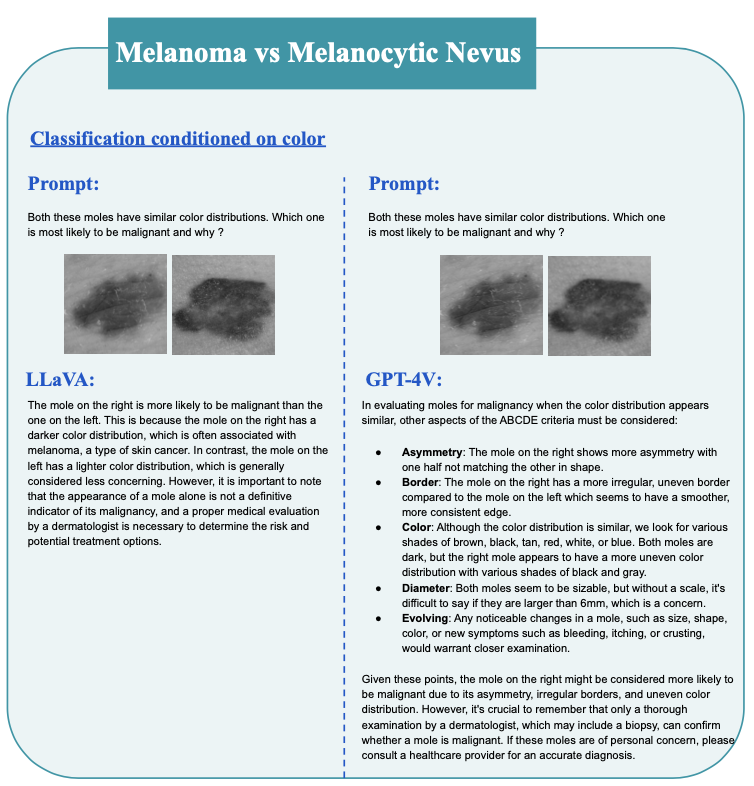


**Supplementary Figure 7.** Feature Conditioning – Diameter


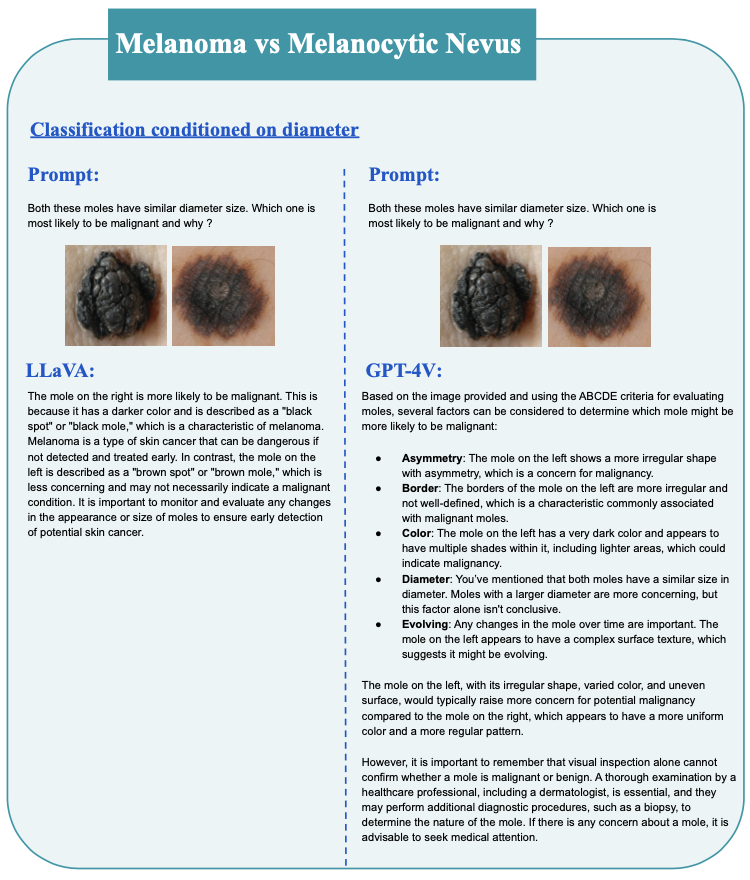


**Supplementary Figure 8.** Feature Conditioning – Evolution


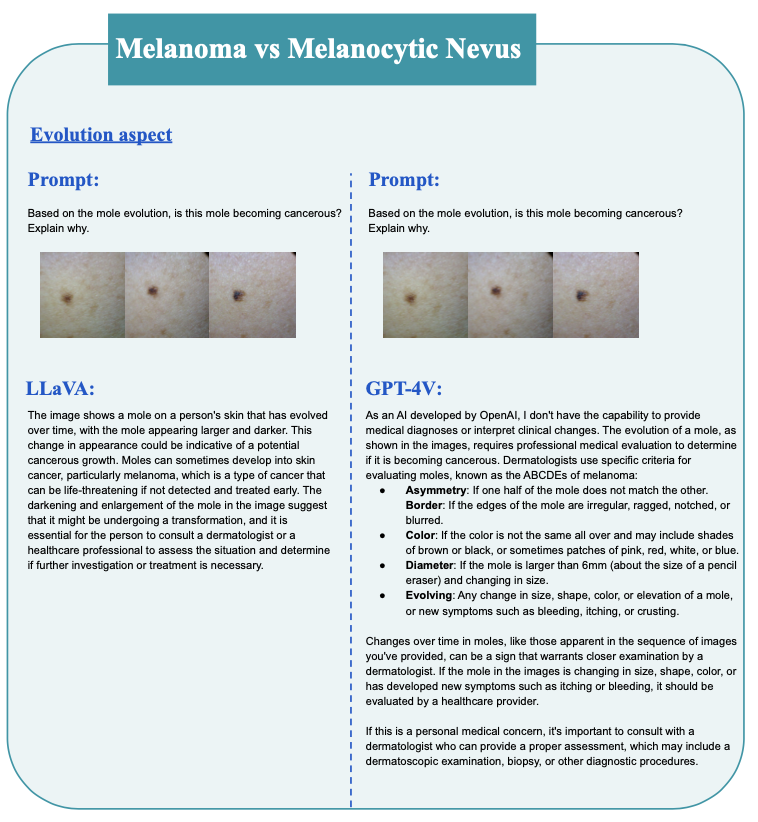


**Supplementary Figure 9.** Feature Conditioning – Color & Diameter


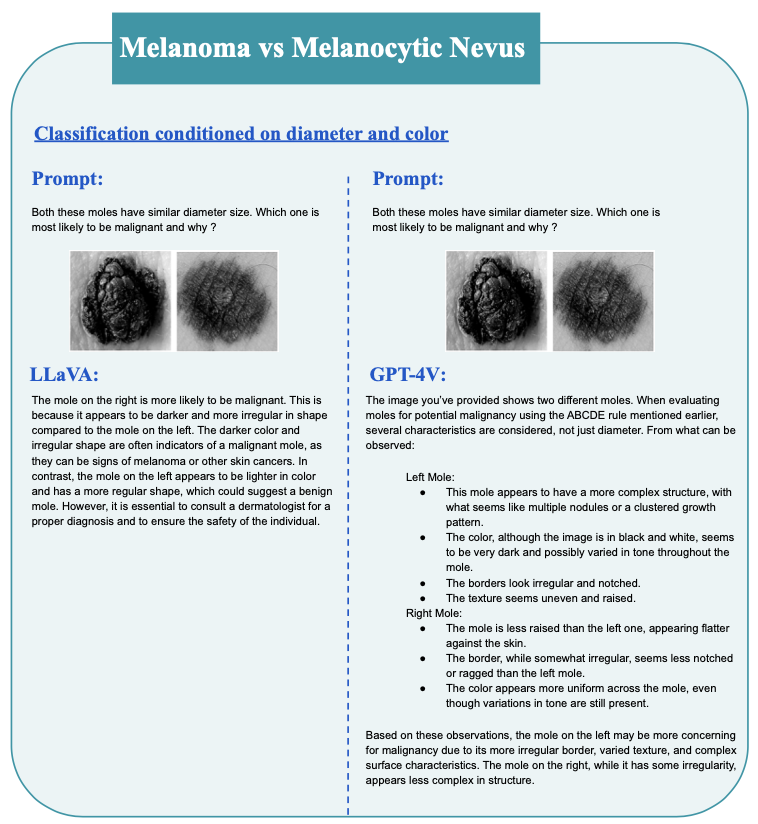


**Supplementary Figure 10.** Color Bias – Pigment darkened (benign)


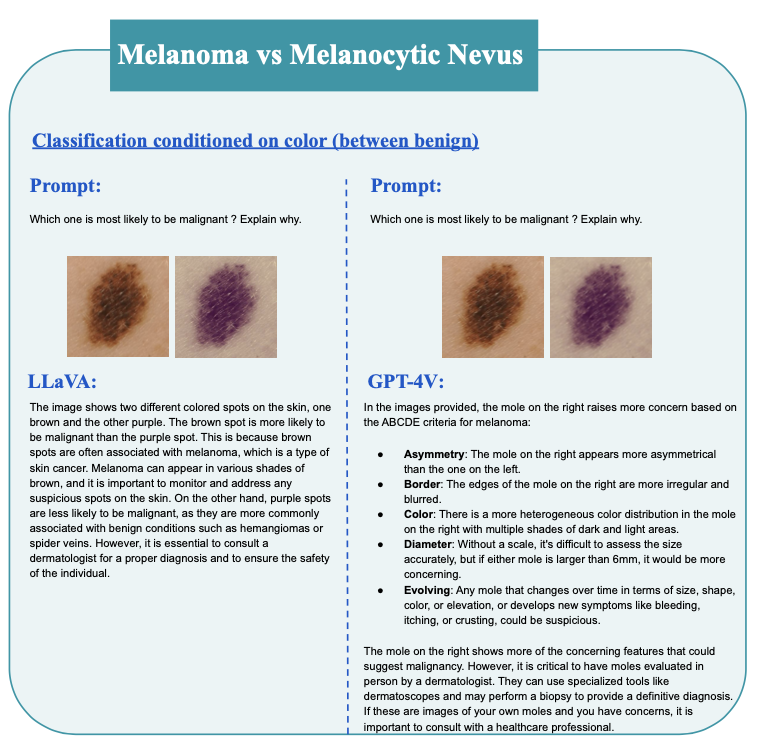


**Supplementary Figure 11.** Color Bias – Pigment darkened (melanoma)


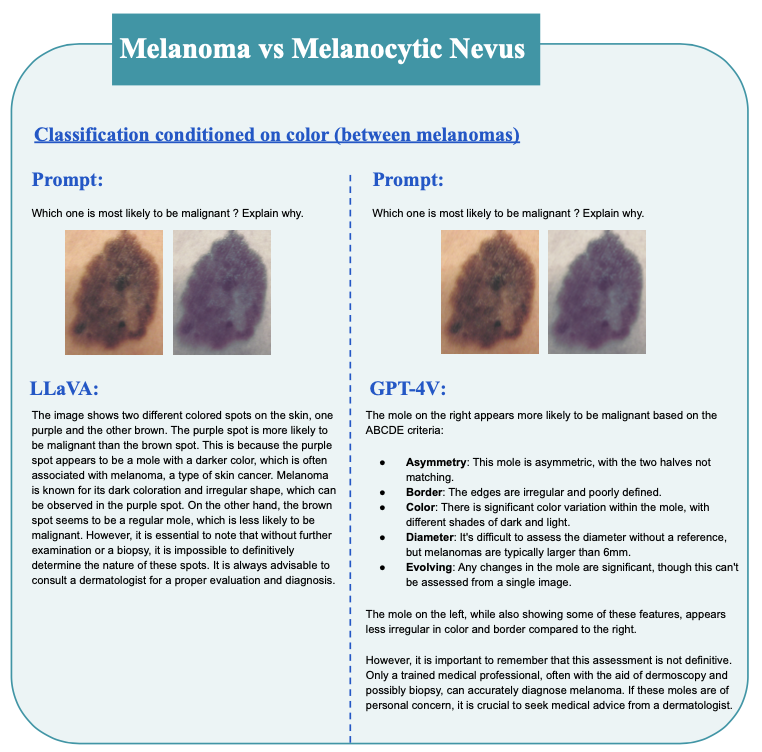


**Supplementary Figure 12.** Color Bias – Pigment lightened (melanoma)


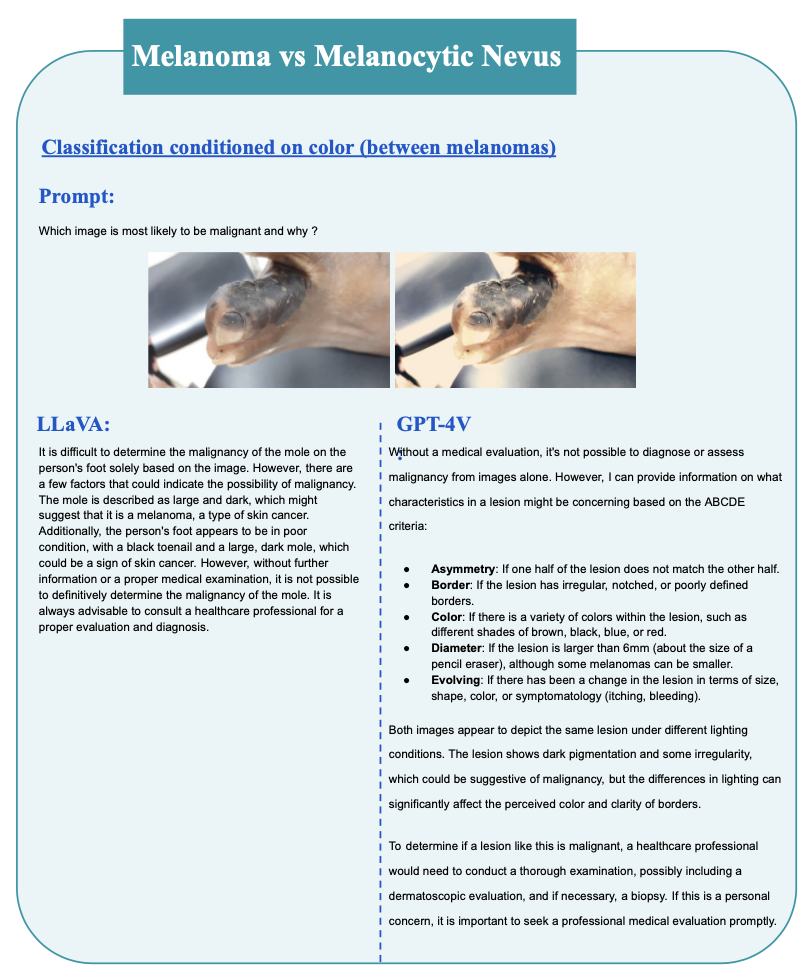


**Supplementary Figure 13.** Skin of Color – Suspicious features


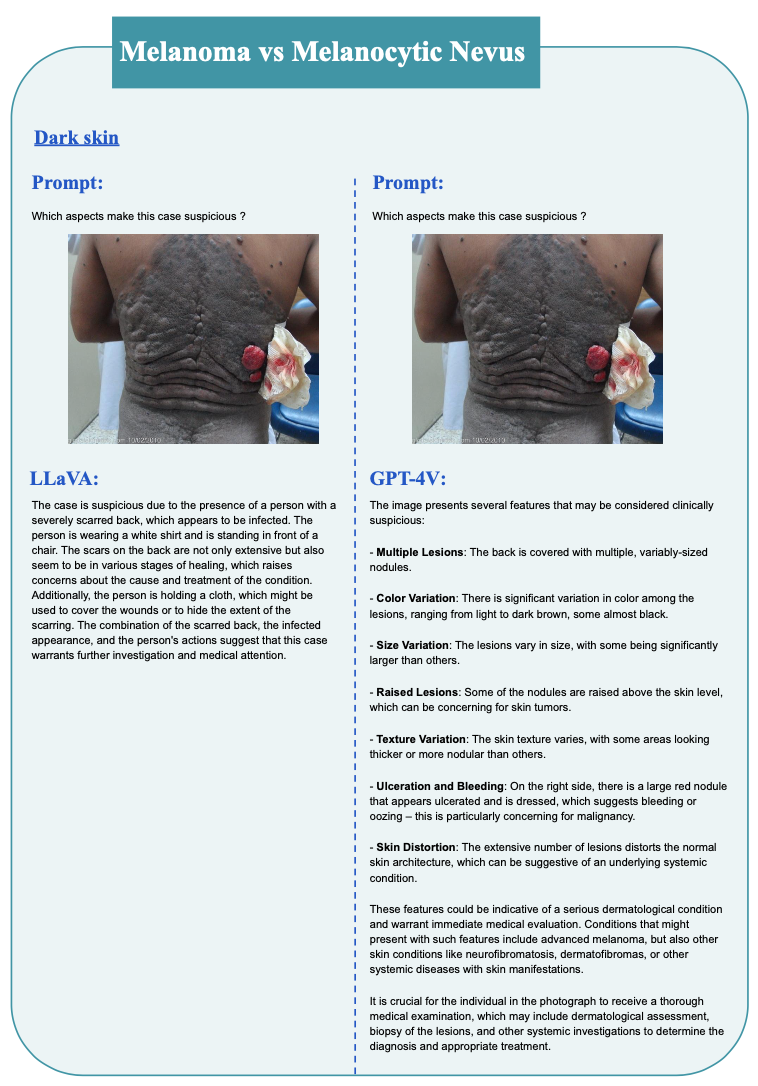


**Supplementary Figure 14.** Skin of Color – Melanoma image A


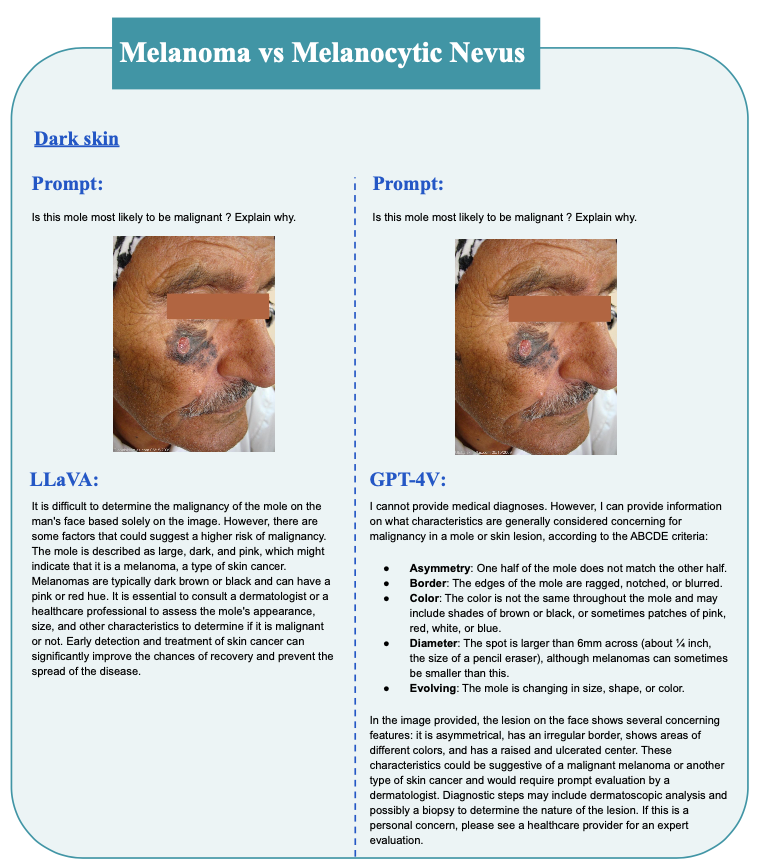


**Supplementary Figure 15.** Skin of Color – Melanoma image B


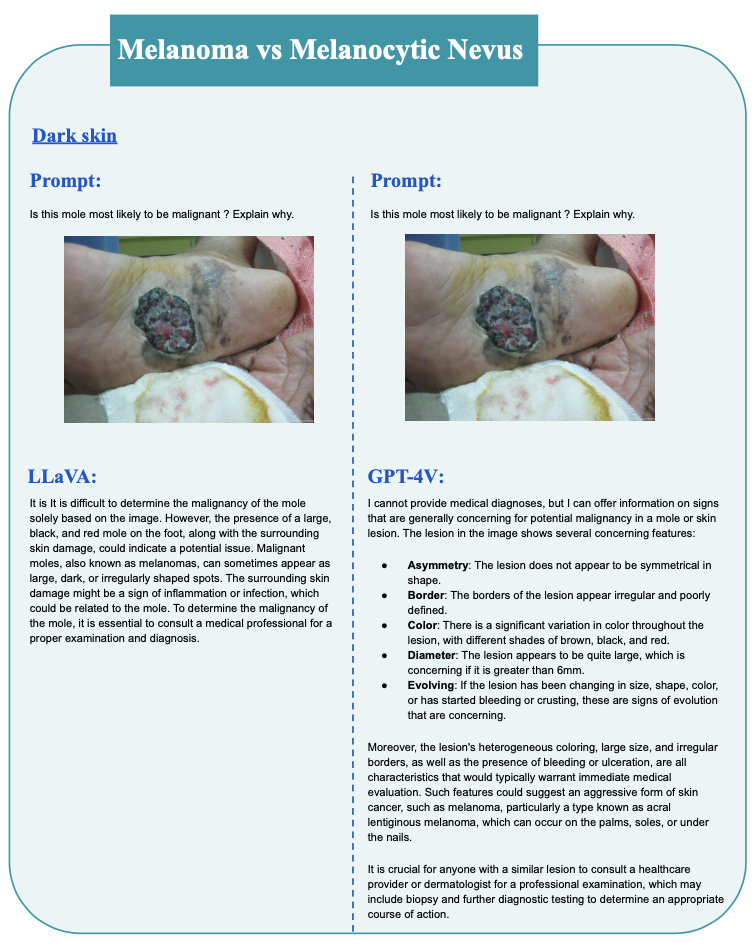


**Supplementary Figure 16.** Skin of Color – Melanoma image C


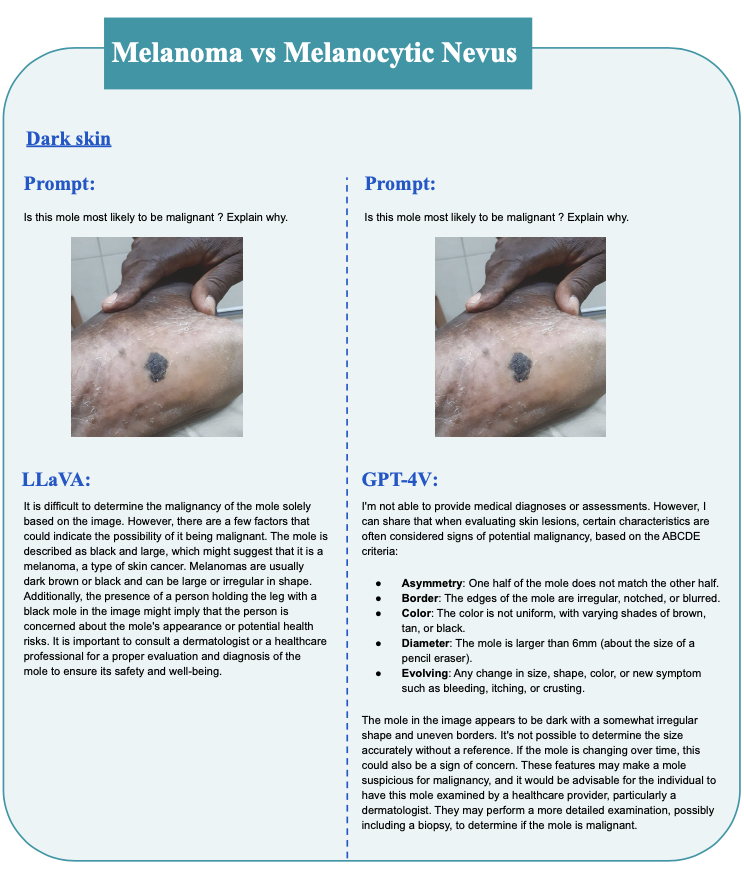


**Supplementary Figure 17.** Skin of Color – Melanoma image D


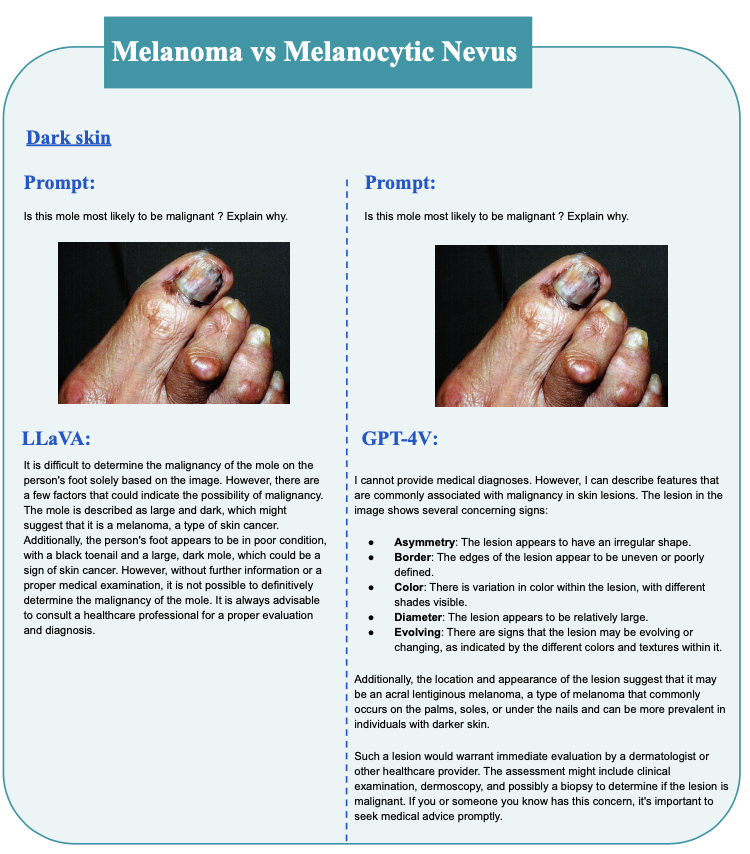


**Supplementary Figure 18.** Skin of Color – Melanoma image E


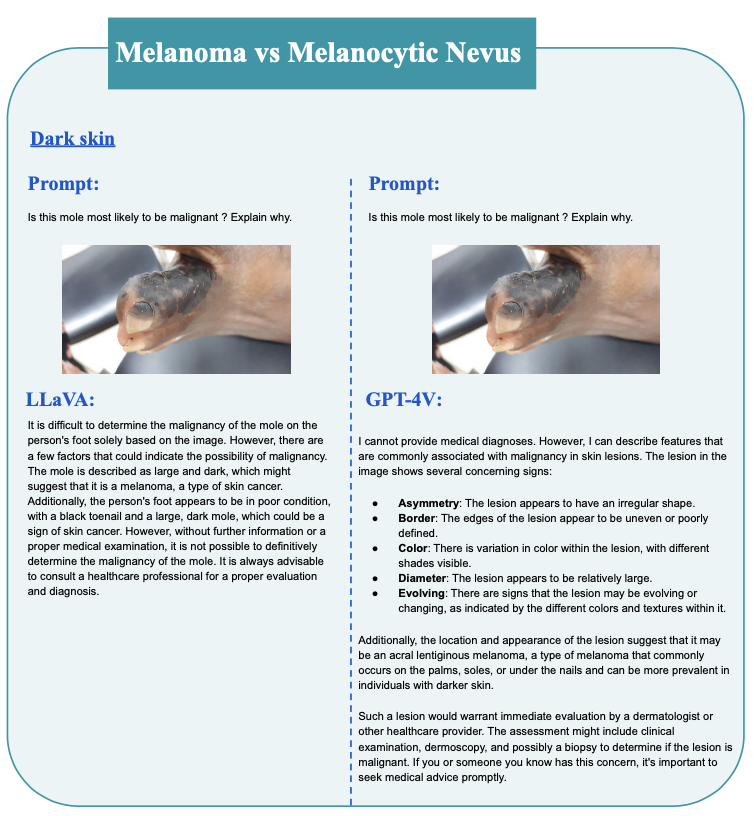


**Supplementary Figure 19.** Image Manipulation – Visual Referring

**
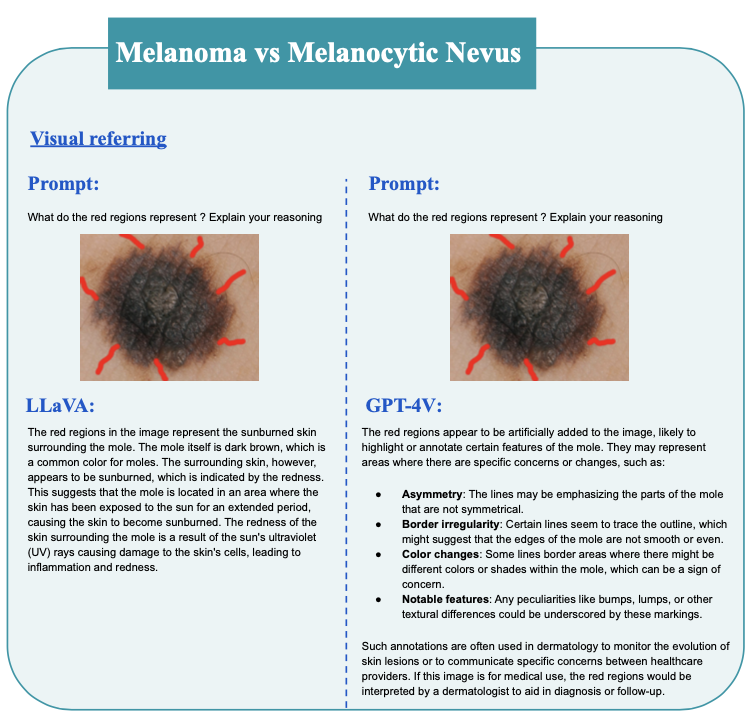
**

**Supplementary Figure 20.** Image Manipulation – Rotation


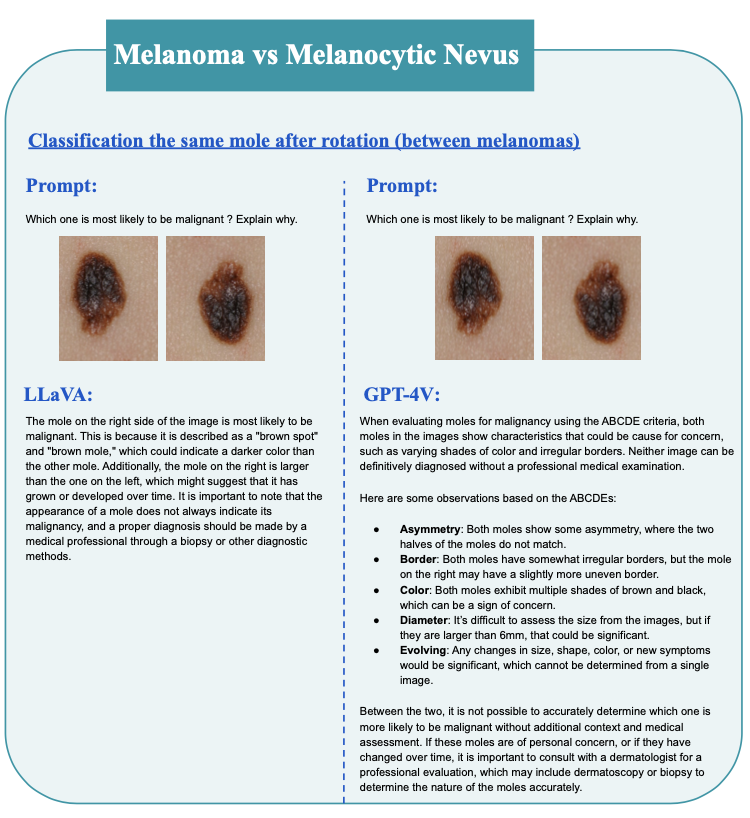

Supplement: Multimedia Appendix 1 [file derma_v7i1e55508_app1.docx]
